# Creating and sharing reproducible research code the workflowr way

**DOI:** 10.12688/f1000research.20843.1

**Published:** 2019-10-14

**Authors:** John D. Blischak, Peter Carbonetto, Matthew Stephens

**Affiliations:** 1 Department of Human Genetics, University of Chicago, Chicago, IL, 60637, USA; 2 Research Computing Center, University of Chicago, Chicago, IL, 60637, USA; 3 Department of Statistics, University of Chicago, Chicago, IL, 60637, USA

**Keywords:** reproducibility, open science, workflow, R, interactive programming, literate programming, version control

## Abstract

Making scientific analyses reproducible, well documented, and easily shareable is crucial to maximizing their impact and ensuring that others can build on them. However, accomplishing these goals is not easy, requiring careful attention to organization, workflow, and familiarity with tools that are not a regular part of every scientist's toolbox. We have developed an R package, **workflowr**, to help all scientists, regardless of background, overcome these challenges. **Workflowr** aims to instill a particular "workflow" — a sequence of steps to be repeated and integrated into research practice — that helps make projects more reproducible and accessible.This workflow integrates four key elements: (1) version control (via **Git**); (2) literate programming (via R Markdown); (3) automatic checks and safeguards that improve code reproducibility; and (4) sharing code and results via a browsable website. These features exploit powerful existing tools, whose mastery would take considerable study. However, the **workflowr** interface is simple enough that novice users can quickly enjoy its many benefits. By simply following the **workflowr** "workflow", R users can create projects whose results, figures, and development history are easily accessible on a static website — thereby conveniently shareable with collaborators by sending them a URL — and accompanied by source code and reproducibility safeguards. The **workflowr** R package is open source and available on CRAN, with full documentation and source code available at https://github.com/jdblischak/workflowr.

## Introduction

A central tenet of the scientific method is that results should be independently verifiable — and, ideally, extendable — by other researchers. As computational methods play an increasing role in many disciplines, key scientific results are often produced by computer code. Verifying and extending such results requires that the code be “reproducible”; that is, it can be accessed and run, with outputs that can be corroborated against published results ^1– 9^. Unfortunately, this ideal is not usually achieved in practice; most scientific articles do not come with code that can reproduce their results ^10– 13^.

There are many barriers to sharing reproducible code and corresponding computational results ^14^. One barrier is simply that keeping code and results sufficiently organized and documented is difficult — it is burdensome even for experienced programmers who are well-trained in relevant computational tools such as version control (discussed later), and even harder for the many domain scientists who write code with little formal training in computing and informatics ^15^. Further, modern interactive computer environments (e.g., R, Python), while greatly enhancing code development ^16^, also make it easier to create results that are irreducible. For example, it is all too easy to run interactive code without recording or controlling the seed of a pseudo-random number generator, or generate results in a “contaminated” environment that contains objects whose values are critical but unrecorded. Both these issues can lead to results that are difficult or impossible to reproduce. Finally, even when analysts produce code that is reproducible in principle, sharing it in a way that makes it easy for others to retrieve and use (e.g., via GitHub or Bitbucket) involves technologies that many scientists are not familiar with ^13, 17^.

In light of this, there is a pressing need for easy-to-use tools to help analysts maintain reproducible code, document progress, and disseminate code and results to collaborators and to the scientific community. We have developed an open source R ^18^ package, **workflowr**, to address this need. The **workflowr** package aims to instill a particular “workflow” — a sequence of steps to be repeated and integrated into research practice — that helps make projects more reproducible and accessible. To achieve this, **workflowr** integrates four key features that facilitate reproducible code development: (1) version control ^19, 20^; (2) literate programming ^21^; (3) automatic checks and safeguards that improve code reproducibility; and (4) sharing code and results via a browsable website. These features exploit powerful existing tools, whose mastery would take considerable study. However, the **workflowr** interface is designed to be simple so that learning it does not become another barrier in itself and novice users can quickly enjoy its many benefits. By simply following the **workflowr** “workflow”, R users can create projects whose results and figures are easily accessible on a static website — thereby conveniently shareable with collaborators by sending them a URL — and accompanied by source code and reproducibility safeguards. The Web-based interface, updated with version control, also makes it easy to navigate through different parts of the project and browse the project history, including previous versions of figures and results, and the code used to produce them. By using **workflowr**, all this can be achieved with minimal experience in version control systems and Web technologies.

The **workflowr** package builds on several software technologies and R packages, without which this work would have been impossible. **Workflowr** builds on the invaluable R Markdown literate programming system implemented in **knitr** ^22, 23^ and **rmarkdown** ^21, 24^, which in turn build on **pandoc**, the “Markdown” markup language, and various Web technologies such as Cascading Style Sheets and Bootstrap ^25^. Several popular R packages extend **knitr** and **rmarkdown** for specific aims such as writing blogs ( **blogdown** ^26^), monographs ( **bookdown** ^27^), and software documentation ( **pkgdown** ^28^). Analogously, **workflowr** extends **rmarkdown** with additional features such as the reproducibility safeguards, and adds integration with the version control system **Git** ^19, 20^. **Git** was designed to support large-scale, distributed software development, but in **workflowr** it serves a different purpose: to record, and provide access to, the development history of a project. **Workflowr** also uses another feature of **Git**, “remotes”, to enable collaborative project development across multiple locations, and to help users create browsable projects via integration with popular online services such as GitHub Pages and GitLab Pages. These features are implemented using the R package **git2r** ^29^, which provides an interface to the **libgit2** C library. Finally, beyond extending the R programming language, **workflowr** is also integrated with the popular **RStudio** interactive development environment ^30^.

In addition to the tools upon which **workflowr** directly builds, there are many other related tools that directly or indirectly advance open and reproducible data analysis. A comprehensive review of such tools is beyond the scope of this article, but we note that many of these tools are complementary to **workflowr** in that they tackle aspects of reproducibility that **workflowr** currently leaves to the user, such as management and deployment of computational environments and dependencies (e.g., **conda**, **Homebrew**, **Singularity**, **Docker**, **Kubernetes**, **packrat** ^31^, **checkpoint** ^32^, **switchr** ^33^, **RSuite** ^34^); development and management of computational pipelines (e.g., **GNU Make**, **Snakemake** ^35^, **drake** ^36^); management and archiving of data objects (e.g., **archivist** ^37^, Dryad ^38^, Zenodo); and distribution of open source software (e.g., CRAN, Bioconductor ^39^, Bioconda ^40^). Most of these tools or services could be used in combination with **workflowr**. There are additional, ambitious efforts to develop cloud-based services that come with many computational reproducibility features (e.g., Code Ocean, Binder, Gigantum, The Whole Tale). Many of these platforms manage individual projects as **Git** repositories, so **workflowr** could, in principle, be installed and used on these platforms, possibly to enhance their existing features. Other R packages with utilities to facilitate reproducibility that could complement **workflowr** include **ProjectTemplate** ^41^, **rrtools** ^42^, and **usethis** ^43^, as well as many of the R packages listed in the “Reproducible Research” CRAN Task View.

Of the available software tools facilitating reproducible research, perhaps the closest in scope to **workflowr** are the R package **adapr** ^44^ and the Python-based toolkit **Sumatra** ^45^. Like **workflowr**, both **adapr** and **Sumatra** use version control to maintain a project development history. Unlike **workflowr**, both place considerable emphasis on managing and documenting dependencies (software and data), whereas **workflowr** only records this information. In contrast, **workflowr** places more emphasis on literate programming — the publishing of text and code in a readable form — and more closely integrates other features such as tracking project development history via **Git** with literate programming.

The **workflowr** R package is available from CRAN and GitHub, and is distributed under the flexible open source MIT license (see *Software availability*). The R package and its dependencies are straightforward to install while being highly customizable for more dedicated users. Extensive documentation, tutorials, and user support can be found at the GitHub site. In the remainder of this article, we describe the **workflowr** interface, explain its design, and give examples illustrating how **workflowr** is used in practice.

## Operation

In this section, we give an overview of **workflowr**’s main features from a user’s perspective. For step-by-step instructions on starting a **workflowr** project, see the “Getting started with **workflowr**” vignette.

**Figure 1.**
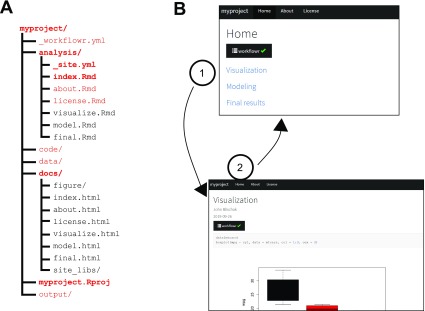
The workflowr package helps organize project files and results. **A**) The function wflow_start() populates a project directory with all the files and subdirectories (shown in red) needed to begin a **workflowr** project. This default directory structure encourages users to organize their files as the project progresses—as the project develops, additional Rmd files may be organized in the "analyses" folder. This is only a suggested structure; users can change the names of most files and directories. Required files are shown in boldface. **B**) All results are organized into a website (all HTML files generated by **workflowr** are automatically stored in docs/). The use of hyperlinks allows for efficient access to the results. The screenshots above illustrate how a **workflowr** website can be navigated. Clicking a hyperlink in the main page, index.html, (1) navigates the browser to a webpage containing some results, visualize.html; clicking on the “Home” hyperlink (2) in the navigation bar brings the browser back to the main page. For larger projects, the navigation bar can be used to quickly access different sections of a project.

For basic usage, only five functions are needed (summarized here, and described in more detail later):

- wflow_start() initializes a new project, including the template directory structure ( Figure 1A);
- wflow_build() renders webpages from R Markdown (Rmd) analysis files, with reproducibility safeguards in place;
- wflow_publish() renders the webpages and updates the project development history—it commits the code, calls wflow_build(), then commits the webpages;
- wflow_status() reports the status of the project files; and
- wflow_git_push() uploads the results from the user’s local repository to a website hosting service.

The primary output of **workflowr** is a project website for browsing the results generated by the Rmd analysis files ( Figure 1B). The use of websites to organize information is, of course, now widespread. Nonetheless, we believe they are under-utilized for organizing the results of scientific projects. In particular, hypertext provides an ideal way to connect different analyses that have been performed, and to provide easy access to relevant external data (e.g., related work or helpful background information); see Figure 1B and *Use cases* below.

### Organizing the project: wflow_start()

The function wflow_start() facilitates project organization by populating a directory with suggested subdirectories, scripts, and configuration files for a data analysis project ( Figure 1A). The subdirectories created by default are analysis/, where the Rmd analysis files are stored; docs/, which stores the website HTML files; code/, which is intended for longer-running scripts, compiled code (e.g., C++) and other source code supporting the data analyses; data/, for storing raw data files; and output/, for saving processed data files and other outputs generated by the scripts and analyses. This setup is flexible and configurable; only two of the directories, analysis/ and docs/, are required, and both can be renamed later.

In addition to creating a default file structure for a data analysis project, wflow_start() also initializes the project development history: it creates a **Git** repository, and commits the files and directories to this repository. This is all done behind the scenes so no familiarity with **Git** is needed. We give more details about the **Git** repository in the *Implementation* section below.

In some cases, a user will have an existing project (with files that may or may not be tracked by **Git**), and would like to incorporate **workflowr** into the project — wflow_start() also easily accommodates this scenario, with additional arguments to control how the **workflowr** files are added to the existing project. See the package vignette, “Migrating an existing project to use **workflowr**,” for more details; it can be accessed by running vignette("wflow-03-migrating") after loading the **workflowr** package in R.

Finally, wflow_start() changes R’s working directory to the root of the project directory. Although this is a simple step, it is important for correctly resolving file paths. Forgetting to change the working directory is a very common source of errors in data analyses.

### Generating results reproducibly: wflow_build()

In a **workflowr** project, analyses are performed using the R Markdown literate programming system ^21^. The user develops their R code inside Rmd files in the analysis/ directory, then calls wflow_build(), which runs the code and renders the results as HTML files in the docs/ directory. The wflow_build() function extends the render_site() command from the **rmarkdown** package with several reproducibility safeguards:

1. It creates a clean R session for executing the code. This is critical for reproducibility—results should not depend on the current state of the user’s R environment, and all objects necessary to run the code should be defined in the code or loaded by packages.

2. It automatically sets the working directory in a consistent manner (the exact setting is controlled by a configuration file; see *Implementation* below). This prevents one of the most common failures to reproduce in R—not setting the working directory before running the R script, resulting in incorrectly resolved relative file paths.

3. It sets a seed for the pseudorandom number generator before executing the code. This ensures that analyses that use random numbers always return the same result.

4. It records information about the computing environment, including the operating system, the version of R used, and the packages that were used to produce the results.

**Figure 2.**
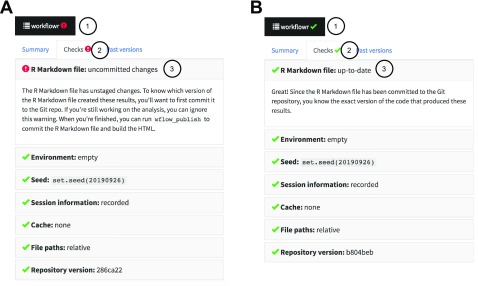
The workflowr reproducibility report summarizes the reproducibility checks inside the results webpage. ( **A**) A button is added to the top of each webpage. Clicking on the button (1) reveals the full reproducibility report with multiple tabs. If any of the reproducibility checks have failed, a red warning symbol (!) is shown. Clicking on the "Checks" tab (2) summarizes the reproducibility checks, with icons next to each check indicating a pass or failure. Clicking on an individual item (3) reveals a more detailed description of the reproducibility check, with an explanation of why it passed or failed. In ( **A**), the Rmd file contains changes that have not yet been committed, so one of the reproducibility checks has failed (uncommitted changes are acceptable during active development, but not acceptable when results are published). In this case, the recommendation is given to run wflow_publish() to fix the issue. ( **B**) If all the **workflowr** reproducibility checks pass, the **workflowr** button shows a green check mark (✔), and clicking an individual item in the reproducibility report (3) gives more detail on the reproducibility check.

Finally, wflow_build() summarizes the results of these reproducibility safeguards in a report at the top of the webpage, along with additional “reproducibility checks”, which alert the user to potential reproducibility issues, such as changes that were not committed to the project development history, and the use of (non-reproducible) absolute file paths ( Figure 2).

### Keeping track of the project’s development: wflow_publish()

As a project progresses, many versions of the results will be generated as results are scrutinized, analyses are revised, errors are corrected, and new data are considered. Keeping track of a project’s evolution is important for documenting progress and retracing the development of the analyses. This is sometimes done without version control tools by copying code and results whenever an important change is made. This typically results in a large collection of files with names such as results-v2-final_final.pdf or anova_analyses_before_adding_new_samples.R. This approach is tedious and error-prone, and makes it difficult to communicate changes to collaborators.

The version control system, **Git**, provides a more systematic and reliable way to keep track of a project’s development history. However, **Git** was designed to manage source code for large-scale software projects, and using it for scientific analyses brings some specific challenges. The relative complexity of **Git** provides a high barrier to entry, discouraging many researchers from adopting it for their projects. And **Git** is not ideally suited to data analysis projects where one wants to coordinate the tracking of source code, data, and the results generated by the code and data. Using **Git** commands to identify the version of the code that was used to generate a result can be non-trivial.

**Figure 3.**
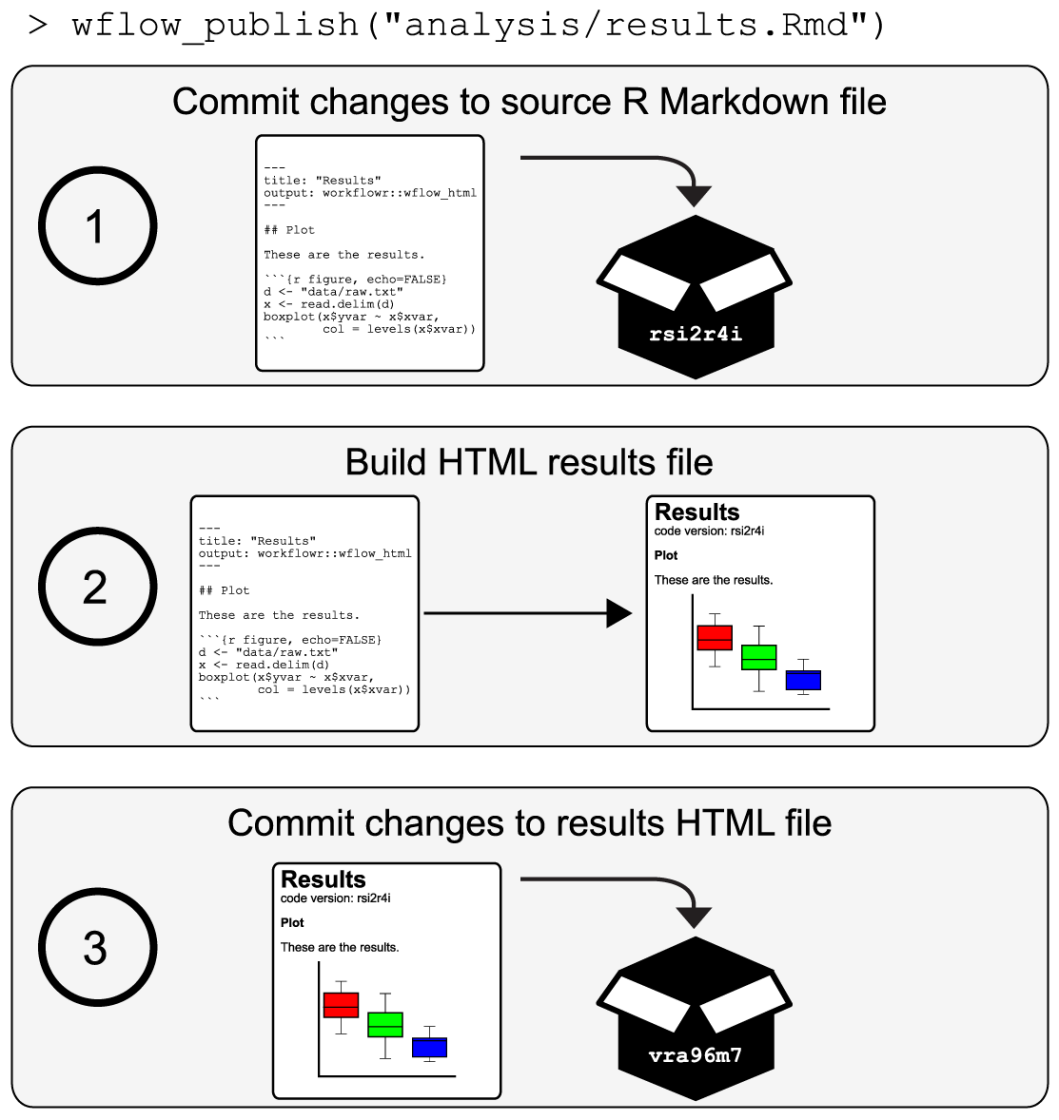
**The function** wflow_publish() simplifies and coordinates tracking of the source code and results files in a Git repository. The function performs a three-step procedure to store the code and results in a project development history, and ensure that the results HTML file is always created from a unique and identifiable versioned Rmd analysis file. (1) The first step commits the changes to the Rmd analysis file. (2) The second step builds the results HTML file from the Rmd file. These two steps ensure that the results were generated from the committed version of the Rmd file. Furthermore, the unique version of the Git repository is inserted directly into the HTML file so that the source code used to generate the results is easily identified and accessed. If the code generates an error, the entire process is aborted and the previous commit made in the first step is undone. (3) The results HTML file, as well as any related figure files, are committed to the Git repository. Thus, the versioning of Rmd analysis files and corresponding HTML results files are coordinated whenever wflow_publish() is used.

The wflow_publish() function is designed to address these challenges: it takes the steps necessary to coordinate tracking of code and results, and reduces these steps to calling a single, easy-to-use function. The command performs three steps, detailed in Figure 3. These steps are designed to ensure that each new collection of results added to the project development history has been produced by a unique and identifiable version of an Rmd analysis file.

Even experienced **Git** users will benefit from using wflow_publish(). Besides the convenience of a single function, wflow_publish() ensures that:

1. Every commit to an (Rmd) analysis file is associated with a commit to the results file generated by that analysis file.
2. An analysis file is only published and committed if it runs successfully; on failure, wflow_publish() aborts, and neither code nor results are committed to the **Git** repository (R code that does not work can still be committed to a **workflowr** project via other methods, e.g., directly using **Git**, but it will not be associated with a committed results file).

Publishing an analysis is not necessarily final — after calling wflow_publish(), the analysis can be repeatedly updated and re-published using wflow_publish(). Each time wflow_publish() succeeds in committing a new version of the code and results, a link to previously published versions of the analysis are embedded in the webpage so that readers can easily access previous versions and compare with the latest results.

### Checking in on the project’s development: wflow_status()

**Figure 4.**
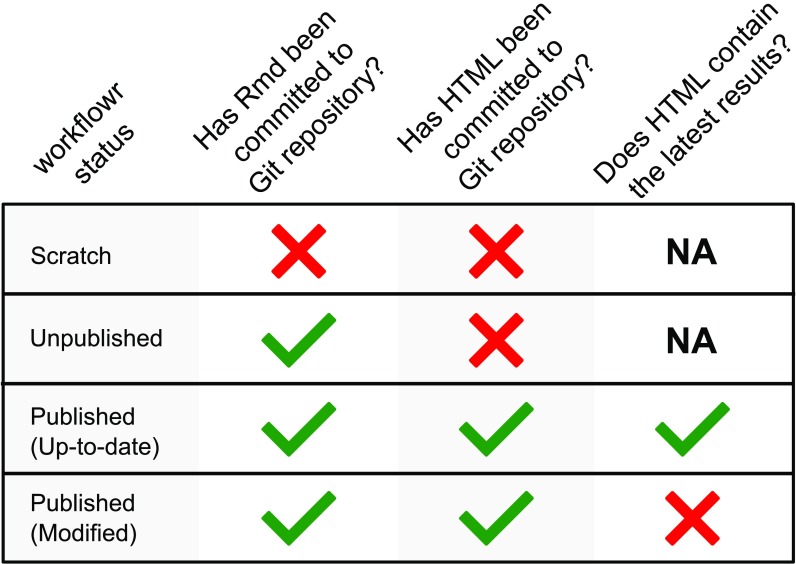
The workflowr package is an R Markdown-aware version control system. The function wflow_status() assigns a state to each Rmd file in the **workflowr** project based on its status in the Git repository’s working tree, and based on the Git status of the associated HTML results file.

As a **workflowr** project grows, it is important to be able to get an overview of the project’s status and identify files that may need attention. This functionality is provided by the wflow_status() command, which gives the status of each Rmd file in the project — either “scratch”, “unpublished”, or “published”, whose definitions are given in Figure 4. The “published” Rmd files, which are those that have been run through wflow_publish(), are further recorded as either “up-to-date” or “modified” depending on whether the Rmd file has been modified since wflow_publish() was run. The wflow_status() function highlights all Rmd files in the “scratch”, “unpublished” or “modified” states, and suggests suitable next steps.

### Sharing code and results: wflow_git_push()

The version-controlled website created by **workflowr** is self-contained, so it can be hosted by most Web servers with little effort. Once the website is available online, the code and results can be shared with collaborators and colleagues by providing them with the website’s URL. Similarly, the **workflowr** repository can also serve as a companion resource for a manuscript by referencing the website URL in the paper.

Since a **workflowr** project is also a **Git** repository, the most convenient way to make the website available online is to use a **Git** hosting service. The **workflowr** package includes functions wflow_use_github() and wflow_use_gitlab() to simplify the setup process on two of the most widely used services, GitHub and GitLab. Once a user has created a **Git** repository on one of these online platforms, the project can be easily uploaded using wflow_git_push() (there is also a companion function wflow_git_pull(), which is used when multiple people are collaborating on a **workflowr** project, or when a project is being updated from multiple computers).

The results files in a **workflowr** website include links to past versions of analysis and figures, making it easy for collaborators to benefit from the versioning of analyses without knowing anything about **Git**. For example, if a collaborator wants to download a previous version of a figure generated several months ago, this can be done by navigating the links on the **workflowr** website.

### Installation

The **workflowr** package is available on CRAN. It works with R versions 2.3.5 or later, and can be installed on any major platform that is supported by R (Linux, macOS, Windows). It is regularly tested on all major operating systems via several continuous integration services (AppVeyor, CircleCI, Travis CI). It is also regularly tested by CRAN using machines running Debian GNU/Linux, Fedora, macOS, Solaris, and Windows.

Because **workflowr** uses the **rmarkdown** package to build the HTML pages, it requires the document conversion software **pandoc** to be installed. The easiest way for R users to install **pandoc** is to install **RStudio**.

Installing **Git** is not required because the R package dependency **git2r** includes **libgit2**, a minimal **Git** implementation (nonetheless, installing **Git** may be useful for occasional management of the **Git** repository outside regular **workflowr** usage).

### Customization

**Workflowr** projects are highly customizable. For example, the look of the webpages can be customized, via options provided by the **rmarkdown** package, by editing the analysis/_site.yml configuration file. Additional settings specific to **workflowr**, such as setting the seed for the pseudorandom number generator, or setting the working directory for the Rmd files, can be controlled in the _workflowr.yml file.

## Implementation

Here we give an overview of the **workflowr** package implementation. All **workflowr** commands can be invoked from R (or **RStudio**) so long as the working directory in R is set to the directory containing a **workflowr** project, or any subdirectory of a **workflowr** project (this is similar to how **Git** commands are invoked). To determine the root directory of a **workflowr** project from a subdirectory, whenever a command is called from the R console, **workflowr** uses the **rprojroot** ^46^ R package to search for the **RStudio** project file stored at the root of the project (the **RStudio** project file is a required file, so if this file is deleted, the **workflowr** commands will not work).

### Organizing the project: wflow_start()

The function wflow_start() populates the project directory using predefined template files (see Figure 1). It uses the **glue** ^47^ R package to insert relevant variables, e.g., the name of the project, directly into the newly created files. When wflow_start() is called with git = TRUE (which is the default), a **Git** repository is created in the project directory, and all newly created or modified files are committed to the repository. If the user has never previously created a **Git** repository on their computer, they may need to first call wflow_git_config() to configure **Git**.

### Generating results reproducibly: wflow_build()

The wflow_build() function generates a responsive website from a collection of Rmd files. Both wflow_build() and wflow_publish() support file patterns, also known as “wildcard expansion”; for example, wflow_build("analysis/*.Rmd") will generate webpages for all the Rmd files in the analysis/ directory.

The wflow_build() function extends the render_site() function from the **rmarkdown** package. The render_site() function in turn builds on the Bootstrap framework to create a responsive website with a navigation bar. This rendering step includes downloading and linking to the required CSS and JavaScript files. Many website settings, such as the labels and URLs included in the navigation bar, can be adjusted in the analysis/_site.yml configuration file (these options can also be set individually inside the Rmd files, which will override the default options set in analysis/_site.yml). Like other R packages that extend **rmarkdown** (e.g., **bookdown**), **workflowr** provides a custom site generator in the function wflow_site(), which alters the website generation process. For example, one change to this process is that the generated website files (the HTML, CSS, JavaScript and figures) are moved instead of copied from analysis/ to docs/. This reduces unnecessary duplication of files. Most of **workflowr**’s key features, including the reproducibility report, are implemented in wflow_html(), which we describe next.

In the **rmarkdown** package, the rendering of individual webpages from Rmd files is controlled by a separate function, html_document(). The **workflowr** package provides an analogous function, wflow_html(). This function also extends html_document(), so all features implemented in **rmarkdown** (e.g., code chunk folding, generating a table of contents from the section headings) are inherited by wflow_html().

Most of the **workflowr** content is added as a preprocessing step prior to executing the R code in the Rmd file. To achieve this, wflow_html() copies the original Rmd file to a temporary directory, incorporates the additional content, then executes the code. The content embedded into the Rmd file includes a code chunk that calls set.seed(), a code chunk toward the end of the file that calls sessionInfo(), and inline HTML tags for elements such as the reproducibility report ( Figure 2) and links to previous versions of figures. There is also a brief postprocessing step to incorporate additional HTML, CSS, and JavaScript elements needed to display the **workflowr** elements added in the preprocessing step. This postprocessing is done when **pandoc** converts the generated markdown to the final webpage.

The process for embedding links to past versions of files — that is, files added to previous commits in a **Git** repository — requires some additional explanation. Links to past versions are included only if the user has set up a remote repository hosted by either GitHub or GitLab. Clicking on a link to a past version of an Rmd file (or figure file) in a Web browser will load a webpage displaying the R Markdown source code (or figure file) as it is saved in the given commit. For past versions of the webpages, we use an independent service raw.githack.com, which displays the HTML file in the browser like any other webpage (this is because GitHub and GitLab only show the raw HTML code). These links will point to valid webpages only after the remote repository (on GitHub or GitLab) is updated, e.g., using wflow_git_push(). In the current implementation, when an Rmd file (and its corresponding HTML file) is renamed, the webpage does not include links to past versions prior to renaming. So renaming files will limit the ability to browse the project development history.

The wflow_html() function allows for considerable customization of the **workflowr** reproducibility report, and other features. The settings in the analysis/_site.yml configuration file are passed to function html_document() in the **rmarkdown** package, whereas the settings in _workflowr.yml are read by wflow_html(); see help(wflow_html) for a full details on all **workflowr** settings that can be customized in this file. For example, the default function used to record the session information at the bottom of each webpage, sessionInfo(), can be overridden by adding the YAML field sessioninfo (e.g., the function from the **devtools** ^48^ package could be used instead by setting sessioninfo: devtools::session_info()).

To execute the code, wflow_build() first creates a new R session to execute the code. This is implemented using the R package **callr** ^49^.

By default, the **rmarkdown** package renders an Rmd file in the directory where the Rmd file is stored; that is, the R working directory is automatically changed to the directory containing the target Rmd file. By default, wflow_html() overrides the behaviour, and instead executes the R code with respect to the root project directory. This default is intended to improve reproducibility by resolving file paths from a consistent reference point. This execution directory can be controlled by the knit_root_dir option, which is set in the _workflowr.yml configuration file. By default, new projects execute the R Markdown code chunks in the root directory. If this setting is not configured, **workflowr** reverts to the **rmarkdown** default. It is also possible to have a different knit_root_dir setting for different files, but this is generally not recommended as it will make the code more difficult to follow.

### Keeping track of the project’s development: wflow_publish()

One of the steps in wflow_publish(), as we have mentioned, is a call to wflow_build(). It also runs **Git** commands to commit the source code and rendered HTML files ( Figure 3). These **Git** commands are executed behind the scenes. We have also implemented many checks and extensive error handling to make sure that the **Git** repository and R environment are in an acceptable state for committing the results. When an issue arises, wflow_publish() attempts to detect the issue as early as possible, then it reverts the **Git** repository to the initial state and, when possible, suggests how to fix the issue. For example, wflow_publish() will stop if any of the files contain conflicts from a previous merge using **Git**.

### Checking in on the project’s development: wflow_status()

The wflow_status() function checks the status of each Rmd file in the project by comparing the state of the file in the **Git** repository’s working tree against the **Git** status of the corresponding HTML file. In **Git** terminology, a “scratch” Rmd file in a **workflowr** project is an uncommitted file in a **Git** repository; “unpublished” means that the Rmd file is committed to the **Git** repository but the corresponding HTML is not; a "published" Rmd file and its HTML file are both committed to the **Git** repository; and a "modified" Rmd file has changes — these changes can be unstaged, staged, or committed — that were made since the last time the corresponding HTML file was committed ( Figure 4).

Using **git2r**, it is mostly straightforward to determine the status of each file. The only complicated step is determining whether published Rmd files have been modified. If all changes to an Rmd file have been committed to the **Git** history, an Rmd file is considered “modified” if it has modifying commits that are more recent than commits modifying the corresponding HTML file.

### Sharing the code and results: wflow_git_push()

To use wflow_git_push(), the remote **Git** repository must first be configured. The user can configure the remotes manually using the git remote subcommand or using wflow_git_remote(). Alternatively, the **workflowr** package provides two functions, wflow_use_github() and wflow_use_gitlab(), that simplify the creation and configuration of remote repositories hosted on GitHub and GitLab. These two convenience functions also add a navigation bar link with the URL of the remote source code repository. The wflow_use_gitlab() function takes the additional step of activating the GitLab Pages by creating a file .gitlab-ci.yml with the proper configuration (GitHub Pages must be set up manually; there is currently no way to automate this via the GitHub API).

## Use cases

**Workflowr** was officially released on CRAN in April 2018. As of September 2019, it has been downloaded from CRAN over 7,000 times, and it has been adopted by many researchers. The most common use cases are 1) documenting research development and including the project website in the accompanying academic paper, and 2) developing reproducible course materials to share with students. Here we highlight some successful examples.

### Repositories for research projects

**Human dermal fibroblast clonality project**

https://davismcc.github.io/fibroblast-clonality

A **workflowr** project accompanying a scientific paper on computational methods for decoding the clonal substructures of somatic tissues from DNA sequencing data ^50^. The webpages describe how to reproduce the data processing and analysis, along with the outputs and plots.

**Characterizing and inferring quantitative cell cycle phase in single-cell RNA-seq data analysis**

https://github.com/jdblischak/fucci-seq

A **workflowr** project supporting a paper on measuring cell cycle phase and gene expression levels in human induced pluripotent stem cells ^51^. The repository contains the processed data and the code implementing the analyses. The full results can be browsed on the website.

**Flexible statistical methods for estimating and testing effects in genomic studies with multiple conditions**

https://github.com/stephenslab/gtexresults

A **workflowr** project containing the code and data used to produce the results from the GTEx data set that were presented in Urbut *et al.* ^52^.

**Investigations on "truncated adaptive shrinkage"**

https://github.com/LSun/truncash

A **workflowr** project created by a Ph.D. student created to keep track of his investigations into controlling false discoveries in the presence of correlation and heteroskedastic noise. This repository illustrates the use of **workflowr** as a scientific notebook — the webpages contain written notes, mathematical equations, source code, and the outputs generated from running the code.

### Repositories for courses

**Stanford STATS 110**

https://xiangzhu.github.io/stanford-stats110

A **workflowr** website for a statistics course taught at Stanford. The website includes working R examples, homework, the course syllabus, and other course materials.

**Single-cell RNA-seq workshop**

https://github.com/crazyhottommy/scRNA-seq-workshop-Fall-2019

A **workflowr** website for a workshop on analysis of single-cell RNA-seq data offered by the Harvard Faculty of Arts and Sciences Informatics group as part of a two-week long bioinformatics course. The R examples demonstrate how to use several bioinformatics packages such as **Seurat** and **msigdbr** to prepare and analyze single-cell RNA-seq data sets.

**Introduction to GIS in R**

https://github.com/annakrystalli/intro-r-gis

A **workflowr** website for a workshop given at the 2018 Evolutionary Biology Conference. The website includes working R demonstrations, setup instructions, and exercises.

## Summary

Our main aim in developing **workflowr** is to lower barriers to open and reproducible code. **Workflowr** provides a core set of commands that can be easily integrated into research practice, and combined with other tools, to make projects more accessible and reproducible. The R package is straightforward to install, easy to learn, and highly customizable.

Since the first official release of **workflowr** (version 1.0.1, released in April 2018), the core functionality has remained intact, and we expect it to remain that way. The core features of **workflowr** have been carefully tested and revised, in large part thanks to feedback and issue reports from the user community. Our next aim is to implement several enhancements, including:

- Create a centralized **workflowr** project website to make it easier for researchers to share and discover **workflowr** projects.
- Provide additional functions to simplify website hosting on other popular platforms such as Netlify and Heroku.
- As **workflowr** projects grow, it becomes increasingly important to document not only the evolution of the code and results over time, but also how the results interrelate with one another. Therefore, we aim to implement syntax that allows file dependencies to be recorded in the Rmd files, and incorporate checking of dependencies as part of the **workflowr** reproducibility safeguards.

As **workflowr** has been used in a variety of settings, we have also uncovered some limitations. Here we report on some of the more common issues that have arisen.

One limitation is that **Git** — hence **workflowr** — is not well suited to tracking very large files. Therefore, large data files must be left out of the project development history, which reduces reproducibility. One possible workaround is to use **Git LFS** (Large File Storage) or related tools that allow large data files to be tracked and stored remotely inside a **Git** repository. This, however, requires considerable expertise to install and configure **Git LFS**, so it is not a satisfactory solution for some **workflowr** users. Also note that sensitive or secure data can be added to a **workflowr** project so long as the storage and access practices meet the data security requirements ( **workflowr** has options to simplify creation and management of projects with security requirements).

Since **workflowr** builds on **Git**, users who already have experience with **Git** can use **Git** directly to manage their **workflowr** projects. This provides additional flexibility, but is not without risk; for example, **Git** commands such as git reset can be used to alter the project development history, and has the potential to break **workflowr**.

Finally, **workflowr** records information about the computing environment used to generate the results, but it does not provide any facilities for replicating the environment. This is an area with many recent software advances — there are many widely used tools for managing and deploying computational environments, from container technologies such as **Docker** to package managers such as Anaconda and **packrat**. We view these tools as being complementary to **workflowr**, and one future direction would be to develop easy-to-use functions that configure such tools for use in a **workflowr** project.

## Data availability

All data underlying the results are available as part of the article and no additional source data are required.

## Software availability

- Software available from: https://cran.r-project.org/package=workflowr
- Source code available from: https://github.com/jdblischak/workflowr
- Archived source code at time of publication: https://doi.org/10.5281/zenodo.3241801 ^53^
- License: MIT
