# Supplementary material for: Creating and sharing reproducible research code the workflowr way
Source: F1000Res. 2019 Oct 14;8:1749. [Version 1] doi: 10.12688/f1000research.20843.1 (PMC6833990; doi:10.12688/f1000research.20843.1)
Supplement: Peer review history

# Peer review: Reviewer response for version 1

**DOI:** 10.5256/f1000research.22923.r55117

**Published:** 2019-11-08

**Authors:** Peter Baker

**Affiliations:** 1 School of Public Health, University of Queensland, Herston, Qld, Australia

The authors introduce an R package that provides an easy way to set up a workflow for data analysis using R and publish results to a web page.

 The workflowr package seems to work well and appears to be widely used.

 What is relatively novel about this package is that while most workflow tools I have seen in R concentrate on setting up a directory structure, syntax files and producing output and reports from analyses, this package adds both the ability to publish the results to web pages and also to set up a Github or Gitlab project repositories with minimal effort.

 The article is clear and well written as are the associated vignettes. The underlying functions in the R are also well written but perhaps could employ more error checking and reporting (see below).

 While I am unlikely to use this approach myself I think it is an excellent approach for new users since it

1. sets up a project skeleton with instructions,
2. encourages users to document their project and workflow right from the start, and
3. also the package provides quite a few helpful vignettes and guides for various scenarios that should be useful for those starting in the area.

However, I do have minor reservations with the approach outlined, including

- for new users, they must not only learn R but also R Markdown which adds an extra level of complexity;
- while it seems necessary in workflowr, users do not really need to use R Markdown files to produce well documented code (see https://rmarkdown.rstudio.com/articles_report_from_r_script.html) but R Markdown seems better suited to reports and articles;
- the workflow is very R Markdown-centric: experienced users may wish to employ R or other software directly or a build system like Make or drake. While this is relatively straight forward outside of the package, e.g. by adding these to the git repository outside of the package and using employing R directly to update intermediate results the article or documentation do not give any details;
- some functions do not appear to be particularly error-proof for new users, e.g. wflow_git_config will overwrite existing settings without checking or even providing a warning although this may conceivably change in future; and
- new users will undoubtedly run into git merge issues and the version I reviewed (1.4.0) did not seem to cater for such eventualities although this appears to have been addressed according to the change log in the latest version (1.5.0).

Minor comments are:

- I am not sure why only some of the software on page 3 is cited when presumably a reader may benefit from the author's recommendations appropriate to the data analysis workflow area rather than tracking down a general reference for themselves;
- while it is good that the potential pitfalls of using git directly or using git reset are addressed on page 11, it may also prove useful for readers to address limitations and potential pitfalls in more depth, perhaps not in the article but with reference to other material or vignettes. For example, users at all levels might benefit from knowing where to get help on merge conflicts, whether users with large data sets should consider databases rather than git or whether typical git workflows like branching would work with this package,
- it would be nice to see how this package compares to other alternatives like drake but admittedly the scope this article is more an introduction to the workflowr package.

In summary, this article is clear and well written. The package is an original contribution to the range of software addressing reproducibility and workflow in data analysis projects.

I confirm that I have read this submission and believe that I have an appropriate level of expertise to confirm that it is of an acceptable scientific standard.

# Peer review: Reviewer response for version 1

**DOI:** 10.5256/f1000research.22923.r55119

**Published:** 2019-11-04

**Authors:** Przemysław Biecek

**Affiliations:** 1 Faculty of Mathematics and Information Science, Warsaw University of Technology, Warsaw, Poland

I like the workflowr package and I like the paper. Nicely written.

 Reproducibility is a big thing and workflowr lowers the entrance barrier for non technical users. This is a huge benefit.

 The package has has already some visibility (judging based on GitHub stars) and is being adopted to various applications (based on examples presented in the paper).

 I recommend to accept the paper.

 Here are some comments that authors may consider:

- **A. **The point that I am missing the most is the comparison against the drake package. These two packages seems to be similar, maybe complementary. Can they be used together? It would be good to show pros and cons/similarities and differences.
- **B. **I like the workflowr package it is a useful tool. What I am missing is the methodology / description of a process / good practices of how the reproducible analysis should look like. This would be very useful for people that look for precise guidelines on how to integrate the workflowr with every day practice. Wet labs researchers are used to "protocols" that directly guide step by step what to do during the analysis. Maybe it would be possible to give such protocols for reproducible research with the workflowr package. 
- Just to give an example, in the Model Development Process ( https://arxiv.org/abs/1907.04461) article there is an overview of phases and tasks shared across model development. In which phases the workflowr or similar tools shall be used? 
- **C. **Authors have mentioned blogdown and bookdown packages. I think that even a closer match to the reproducibility problem is the package modelDown (see https://joss.theoj.org/papers/10.21105/joss.01444 or GitHub http://github.com/ModelOriented/modelDown). ^1^
  The modelDown package takes predictive models and creates a HTML website with information about session info, binary models, training/test data and model explanations. The website is created without any additional effort. ModelDown automates the most boring part of the modeling i.e. model documentation.
- **D. **When mentioning tools for archivisation of binary objects, it may be also useful to add the pins package recently developed by RStudio (https://cran.r-project.org/web/packages/pins/index.html). It is more limited than other mentioned packages (do not keep information about meta data) but quickly gains popularity.
- **E. **After the "Unfortunately, this ideal is not usually achieved in practice; most scientific articles do not come with code that can reproduce their results" maybe authors could share their thoughts why it is the case. It will be useful to list specific reasons why reproducibility fails. Is it primarily because we do not have proper software, or they software is too complex, or one needs to pay for the proper software, or researchers are not aware of the problem?

I confirm that I have read this submission and believe that I have an appropriate level of expertise to confirm that it is of an acceptable scientific standard.

## References

[1] : modelDown: automated website generator with interpretable documentation for predictive machine learning models. *Journal of Open Source Software*.2019;4(38) : 10.21105/joss.01444 10.21105/joss.01444

# Peer review: Reviewer response for version 1

**DOI:** 10.5256/f1000research.22923.r55176

**Published:** 2019-10-31

**Authors:** Peter F. Hickey

**Affiliations:** 1 Epigenetics and Development Division, Walter and Eliza Hall Institute of Medical Research, Parkville, Vic, Australia

The workflowr package, available from CRAN for 1.5 years, has already demonstrated itself to be a valuable contribution to improving the reproducibility of scientific analyses. This paper does a thorough job setting out the rationale, design, and implementation of the workflowr package. It is clear that the authors have spent considerable time thinking about some of the key challenges of this endeavour, learning about best practises, getting feedback from users, and implementing these using 4 key technologies/features: 

1. Version control.
2. Literate programming.
3. Automatic checks and safeguards to improve code reproducibility.
4. Sharing code and results via a website.

The paper is clearly written and I am happy to approve the article in its current form.

 Some minor comments, queries, and corrections are given below:

- Figure caption 1: '"analyses" folder' is '"analysis" folder' in the figure.
- p5: Re workflowr executing code in a clean session. My impression was that rendering Rmarkdown documents, at least when done by clicking the 'knit' button in RStudio, was already run in a separate process. But I may be mistaken and perhaps this is different from running `rmarkdown::render_site()`?
- Regarding published sites. Not all scientific analyses can be made public, particularly in the early stages. Some discussion of options available for private/protected hosting would be valuable.
- I'm a little wary of relying on raw.githack.com for hosting past versions of the webpages. For example, what if the service becomes unavailable? Does or could workflowr support other hosting services?
- p11: Is the idea of a 'centralized workflowr project website' like that of the homepage https://bookdown.org/ or for an organisation/user to share their personal workflowr projects?

I confirm that I have read this submission and believe that I have an appropriate level of expertise to confirm that it is of an acceptable scientific standard.
